# Assessment of Visual Diagnosis by Podiatrists for HPV and Onychomycosis: The Need for Complementary Tests

**DOI:** 10.3390/jof8020135

**Published:** 2022-01-29

**Authors:** Alberto Aldana-Caballero, Raquel Mayordomo, Félix Marcos-Tejedor

**Affiliations:** 1 Department of Nursing, Physiotherapy and Occupational Therapy, Faculty of Health Sciences, Universidad de Castilla-La Mancha, 45600 Talavera de la Reina, Spain; alberto.aldana@uclm.es; 2 DEDAP Research Group, Department of Anatomy, Cellular Biology and Zoology, Universidad de Extremadura, 10600 Plasencia, Spain; 3 DEDAP Research Group Collaborator, Department of Medical Sciences, Universidad de Castilla-La Mancha, 45600 Talavera de La Reina, Spain; felix.marcostejedor@uclm.es

**Keywords:** onychomycosis, plantar warts, misdiagnosis, complementary tests

## Abstract

Onychomycosis and human papillomavirus (HPV) plantar warts are common in clinical practice. Clinical diagnosis is based on searching for pathognomonic signs and symptoms. However, due to misdiagnosis, podiatrists may unnecessarily prescribe antifungal treatments or burn lesions with chemical agents. The objective of this study was to assess podiatrists’ visual diagnosis of these infections and their willingness to use complementary tests. A 12-item questionnaire was developed to assess visual diagnostic ability. The diagnoses of all lesions were verified before the questionnaire was sent out. The respondents were 415 podiatrists with a range of years of experience. While 86.3% of podiatrists considered complementary tests for onychomycosis necessary, only 21.4% used them regularly. As many as 60.2% would leave a distal subungual onychomycosis untreated based on visual diagnosis. In the case of HPV, only 14.5% of respondents considered complementary tests necessary, although 76.6% would treat a non-HPV lesion with regular chemical agents. Years of experience did not affect the percentage of misdiagnoses. Complementary tests are needed in clinical practice to avoid unnecessary treatments. Podiatrists easily identify clear signs and symptoms but have difficulty making differential diagnoses. Research should focus on ensuring complementary tests are available to health professionals.

## 1. Introduction

Dermatological infections are common problems in clinical practice. Onychomycosis has a prevalence of around 5.5% in the general population and it is more common in people with tinea pedis, estimated in some populations to be as much as 50–80% [1,2]. Normally, 50% of toenail problems found in clinical practice are fungal infections [1,2]. Alongside fungal infections, plantar warts have an annual prevalence of 14%. Patients seek medical care to eliminate symptoms, relieve pain, and offset concerns about infecting other members of the family unit [3].

Other pathological entities present signs and symptoms that hinder differential diagnosis by health professionals. Onychomycosis shares some signs and symptoms with psoriasis, trauma, and lichen planus [2,4], and plantar warts produced by HPV infection can easily be misdiagnosed as hyperkeratosis, calluses, molluscum contagiosum, angiokeratoma, verrucous carcinoma, and other lesions [3,5,6,7,8].

Because clinical diagnosis based on signs and symptoms is difficult in some cases, complementary tests are helpful. For onychomycosis, the diagnostic gold standard is culture [9], but human papillomavirus (HPV) lesions causing plantar warts have no such standard. The diagnosis options available include histopathological examination, ultrasound, dermatoscopy, and PCR. However, the main disadvantages of these methods are that some of them use punch biopsies, with the consequence of wreaking havoc on healthy skin, or imaging that requires specialist training and can be subjective [6,10,11]. Without tests to confirm diagnosis, health professionals may implement treatment empirically, unnecessarily burning healthy skin or prescribing antifungal drugs. The result is unsatisfactory treatment that may lead to antifungal resistance. The same consequences can also stem from misdiagnosis [4,12].

The main objective of this study is to assess podiatrists’ visual diagnostic ability to differentiate between onychomycosis, plantar warts, and similar problems as a way to highlight the need to conduct complementary tests and collate information about podiatrists’ willingness to request complementary tests and how often they do so.

## 2. Materials and Methods

A 12-item visual assessment questionnaire was developed. Six items were intended to assess visual diagnostic ability for onychomycosis and similar pathological entities, and the other six to assess plantar warts and similar foot lesions. The questionnaire was sent to respondents who were licensed podiatrists at 17 Associations of Official Podiatrist, following the inclusion criteria. Onychomycosis was confirmed beforehand by culture or PCR following the diagnostic method recommended in the literature [3,9], and plantar warts were confirmed by histopathological examination or reference material provided for research purposes for the questionnaire.

**Figure 1:**
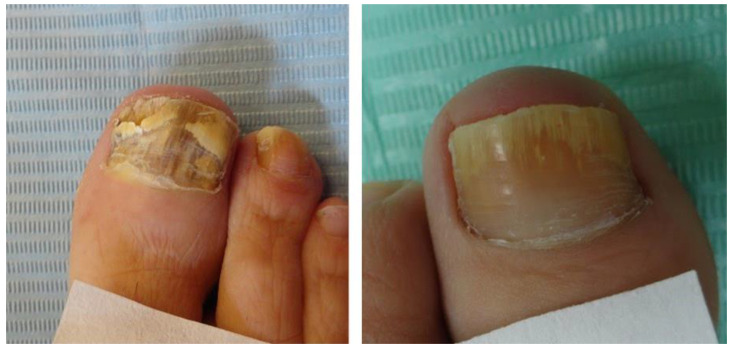
Item 1 and 2: Total dystrophic onychomycosis and onycholysis, respectively.

**Figure 2:**
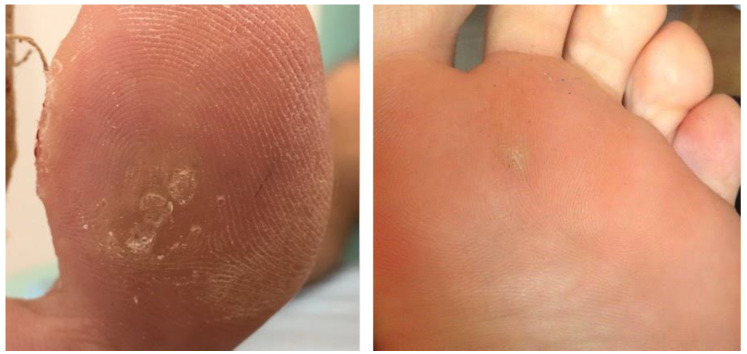
Item 3 and 4: HPV plantar wart on first toe and metatarsal heloma, respectively.

The items included a full spectrum of difficulty, ranging from lesions that were easily differentiable and identifiable by their signs and symptoms (some considered pathognomonic) to lesions showing the same signs and symptoms and therefore requiring a differential diagnosis. In accordance with the prevalence of cases in clinical practice reported in the literature [1,2], 50% of the items for onychomycosis were positive, and differential diagnosis was required with psoriasis, trauma, and onycholysis. The lesions included alongside plantar warts were molluscum contagiosum, dermal neuroma, fibrokeratoma, and heloma. Figure 1 and Figure 2 show four of the items for visual diagnostic assessment from the questionnaire. The complete questionnaire can be viewed in the Supplementary Materials. Blurred images are licensed for research purposes only, not publication.

The questionnaire was filled in by practicing podiatrists and recent podiatry graduates to obtain data on whether years of experience (<10 years, 11–20 years, and >20 years) affected the accuracy of visual diagnosis. Respondents answered the questionnaire only once, and the results were stored in databases for later analysis using SPSS Statistic Software version 22.0 (Armonk, NY, IBM Corp., accessed on 28 January 2022). A descriptive analysis was made, and Pearson’s Chi-square (χ^2^) test was used in contingency tables for the variables of years of experience (<10 years, 11–20 years and >20 years) and correct or incorrect diagnosis, with 95% confidence intervals. Sample size was calculated with a 95% confidence level and a margin of error of 0.05 in a population size of 7817, according to the Spanish National Statistics Institute (2018) [13], with a result of 367.

## 3. Results

The total respondents were 415 podiatrists, 96.6% of whom currently work in this profession in clinical practice.

### 3.1. Consideration of the Need for Complementary Tests

Complementary tests to diagnose onychomycosis were considered necessary by 86.3% of respondents, although 66.3% said they requested them only occasionally. In the case of plantar warts, 85.5% of the respondents did not consider complementary tests necessary (Table 1).

The complementary test of choice for diagnosing onychomycosis is culture (74.3%), followed by PCR (polymerase chain reaction) (10%), and biopsy (histopathological studies) (4.4%), while 0.64% of respondents said they used or had used the Diafactory Test (commercial immunochromatographic assay), and 0.64% requested or used KOH.

For plantar warts, the tests of choice among podiatrists who used complementary tests in some cases were biopsy (histopathology) in 15.5% of cases and PCR in 4.8% of cases.

### 3.2. Visual Diagnostic Assessment

According to the results, 22.7 to 35.7% of respondents would prescribe an antifungal treatment for nail alterations caused by onycholysis, psoriasis, or trauma, while 60.2% of respondents would not treat the most prevalent onychomycosis (distal subungual onychomycosis (DSO)), 10.1% would not prescribe treatment for severe onychomycosis, and 12.8% would not prescribe treatment for superficial white onychomycosis.

In the case of non-plantar wart lesions, 76.6% of respondents would treat using traditional options (chemical burning) and 17.6% would leave a plantar wart untreated due to incorrect diagnosis.

The results of the visual diagnostic assessment (correct and incorrect diagnosis) are shown in Table 2 and Table 3.

### 3.3. Impact of the Experience in Practice to Visual Diagnosis

Years of experience showed no impact on the outcome of visual diagnosis for onychomycosis. However, differences were observed in years of experience between groups for items four and five for plantar lesions (*p* = 0.03 and *p* = 0.025, respectively). The results of years of experience and frequency of misdiagnosis are shown in Table 4 and Table 5, respectively.

## 4. Discussion

This study examines whether podiatrists consider complementary tests necessary to support or verify the diagnosis of onychomycosis or plantar warts, and identifies how often they use the tests available to them. The results show that podiatrists do not normally request complementary tests to confirm foot infections. In the case of onychomycosis, 86.3% of podiatrists consider it necessary to rely on complementary tests to properly diagnose infections, although this result does not concur with frequency of use, as only 21.4% of respondents use them regularly. In other fields, the literature shows that microbiological studies are requested by 3.4% of general physicians and 39.6% of dermatologists [12].

Culture is considered a gold standard in onychomycosis diagnosis, and podiatrists are aware of this according to our results, but this method has disadvantages. It requires correct sample handling to avoid contamination, the procedure is lengthy and may not always be sensitive enough [14], and it has a 40% false negative rate. The KOH test is less used, probably because it is subject to experimental bias, even though it is a rapid, easy technique [2,15].

Molecular methods are now the basis of diagnostic tests for medical clinical practice, but they are not used widely enough to confirm onychomycosis diagnoses, even though methods to detect the presence of fungi from a nail fragment have been described [2,15,16]. PCR is a more sensitive, specific, and rapid method to obtain a result, but only 10% of podiatrists said they had used it at some time.

In our sample, some respondents reported the use of new commercial tests that are available to professionals, e.g., the Diafactory Test (0.64%), but these tests do not detect the vast array of microorganisms capable of producing onychomycosis, such as non-dermatophytic fungi, yeasts, and molds, which have started to overtake fungal infections recently due to new lifestyles and immunodeficiencies in society [2].

Studies of nail histopathology are used by 4.4% of the respondent podiatrists. The disadvantage of these methods is that they cause a lesion that may not warrant the result obtained [17,18]. Benefit−risk balance has to be taken into account, explaining why nail histopathology is the least used method and was reported only by 0.64% of respondents who use the Diafactory Test.

In the case of HPV plantar warts, however, consideration of the need for complementary tests is in line with actual use. Only 12.7% of respondents consider it necessary to rely on complementary tests for the diagnosis of this infection, while 17% said they have used biopsy in some cases. The use of PCR for HPV diagnosis is almost anecdotal, although the literature shows that this method remains the subject of research but lacks implementation in the clinical setting for plantar warts [11,19]. However, it has been studied as a method worth exploring for use as a diagnostic tool for plantar warts [20].

Podiatrists do not typically use complementary tests for either of the infections included in this study, although this finding is in contrast with the results obtained in the assessment of visual diagnostic skills. While 85.5% of respondents do not consider it necessary to use any diagnostic method for HPV plantar warts, 76.6% (Table 3, in bold) would treat a non-HPV lesion with the usual lytic method of the epidermis for plantar warts [21], performing unnecessary chemical burning that causes discomfort to the patient. This result is also supported in items four and six, in Table 3.

The study shows that a third of the respondents (35.7%, in bold, Table 2) prescribe antifungal treatment for a non-fungal nail disorders. This is not only inefficient for treating the problem and frustrating for patients and professionals alike, but also increases the chances of drug resistance. An example of this highlighted in the literature is the growing resistance to Terbinafine, which is obliging podiatrists and health professionals to prescribe other antifungal drugs [22]. It is therefore essential to have appropriate diagnostic tools to combat drug resistance in the treatment of fungal infections, as indicated by Perlin et al. [23], especially in *Candida* species, which currently account for 50% of fungal nail infections [2,24]. The need to use proper diagnostic tools is supported in our results, as item five in Table 2 shows that nearly two thirds (60.2%, in bold, Table 2) of the respondents would leave a fungal nail infection untreated based solely on visual diagnosis.

Years of experience in clinical practice do not appear to affect the accuracy of the visual diagnosis of onychomycosis (Table 4). We can therefore conclude that the percentage of misdiagnosis is generalized, although we found two differences between groups for plantar warts, one of which involved an actual case of plantar warts (*p* = 0.025). This may have been a one-off result, or further research may be needed with more items about HPV infections. The workplace of health professionals could be a variable worth assessing in future studies, but this aspect was not addressed here.

Overall, the results show that it is important for health professionals, especially podiatrists, to have efficient and appropriate diagnostic tools to rely on after their first diagnostic judgment, both for HPV infections and onychomycosis. In light of the results obtained, further research could address the development of diagnostic tools, examine the methods currently available and identify others not yet implemented, working to define methods for use as an important everyday tool when patients present with these infections in clinical practice.

## 5. Conclusions

The podiatrists who answered the questionnaire had a good concordance rate when the lesions showed clear signs and symptoms, but had difficulty establishing differential diagnoses. They are generally willing to use complementary tests, but do not use them enough, despite the rate of misdiagnosis. Research should aim to ensure diagnostic tools are available to health professionals so they can establish appropriate protocols and strategies to use these tools for the ultimate benefit of patients.

## Figures and Tables

**Table 1:** Consideration of the need for complementary tests to diagnose onychomycosis and plantar warts.

	Consider Complementary Tests Necessary for Diagnosis		
	Never Used	Occasionally Used	Always Used
Onychomycosis (86.3%)	12.3%	66.3%	21.4%
HPV plantar warts (14.4%)	81.4%	17.8%	0.7%

**Table 2:** Visual diagnostic assessment for the diagnosis of onychomycosis (correct and incorrect diagnoses).

Correct Diagnosis	Would Treat as Onychomycosis	
	Yes	No
1. Total dystrophic onychomycosis (TDO) (+)	89.9%	10.1%
2. Onycholysis (−)	35.7%	64.3%
3. Superficial white onychomycosis (SWO) (+)	87.2%	12.8%
4. Trauma (−)	22.7%	77.3%
5. Distal subungual onychomycosis (DSO) (+)	39.8%	60.2%
6. Psoriasis (−)	33%	10.1%

**Table 3:** Visual diagnostic assessment for the diagnosis of HPV plantar warts (correct and incorrect diagnoses).

Correct Diagnosis	Would Treat as HPV Plantar Warts	
	Yes	No
1. Fibrokeratoma (−)	22.9%	77.1%
2. Molluscum contagiosum (MC) (−)	76.6%	23.4%
3. Plantar wart (+)	82.4%	17.6%
4. Dermal neuroma (−)	68.4%	31.6%
5. Plantar wart (+)	93%	7%
6. Heloma (−)	37.8%	62.2%

**Table 4:** Years of experience and frequency of misdiagnosis of onychomycosis.

	Diagnosis	Experience (in Years) (n = 415)			Pearson’s χ^2^
		<10 (n = 201)	11–20 (n = 128)	>20 (n = 86)	
TDO	Incorrect	17	13	12	0.368
	Correct	184	115	74	
Onycholysis	Incorrect	76	41	31	0.564
	Correct	125	87	55	
SWO	Incorrect	24	17	12	0.877
	Correct	177	111	74	
Trauma	Incorrect	48	29	17	0.748
	Correct	153	99	69	
DSO	Incorrect	120	79	51	0.917
	Correct	81	49	35	
Psoriasis	Incorrect	72	41	24	0.409
	Correct	129	87	62	

**Table 5:** Years of experience and frequency of misdiagnosis for plantar warts.

	Diagnosis	Experience (in Years) (n = 415)			Pearson’s χ^2^
		<10 (n = 201)	11–20 (n = 128)	>20 (n = 86)	
Fibrokeratoma	Incorrect	45	34	16	0.386
	Correct	156	94	70	
MC	Incorrect	163	89	66	0.054
	Correct	38	39	20	
Plantar wart	Incorrect	37	23	13	0.791
	Correct	164	105	73	
Dermal neuroma	Incorrect	150	79	55	0.03
	Correct	51	49	31	
Plantar wart	Incorrect	21	4	4	0.025
	Correct	180	124	82	
Heloma	Incorrect	77	46	34	0.852
	Correct	124	82	52	

## Data Availability

Not applicable.

## Supplementary Materials

The following supporting information can be downloaded at: https://www.mdpi.com/article/10.3390/jof8020135/s1: Questionnaire used.
