# Supplementary material for: Assessment of Visual Diagnosis by Podiatrists for HPV and Onychomycosis: The Need for Complementary Tests
Source: J Fungi (Basel). 2022 Jan 29;8(2):135. doi: 10.3390/jof8020135 (PMC8878088; doi:10.3390/jof8020135)
Supplement: Supplementary file 1 [file jof-08-00135-s001.zip › jof-1551573-supplementary.pdf]

# Assessment of visual diagnosis in podiatry: Onychomycosis and plantar warts.

Currently, infectious pathology represents one of the main reasons why patients come to the clinic. The diagnosis of them often consists of a clinical assessment of signs and symptoms, however, these pathologies are not exempt from diagnostic failures, and therefore in the following questionnaire, we intend to observe the visual diagnosis of onychomycosis and pathology of skin lesions caused by human papillomavirus in professionals and future professionals of podiatry.

*Mandatory

1. Year obtaining the degree in Podiatry *

*Select only one option.*

- 1988
- 1989
- 1990
- 1991
- 1992
- 1993
- 1994
- 1995
- 1196
- 1997
- 1998
- 1999
- 2000
- 2001
- 2002
- 2003
- 2004
- 2005
- 2006

- 2007
- 2008
- 2009
- 2010
- 2011
- 2012
- 2013
- 2014
- 2015
- 2016
- 2017
- 2018
- 2019

2. Do you currently practice podiatry? *

*Select only one option.*

- Yes
- No

## Onychomycosis assessment

3. Do you consider that complementary laboratory tests are necessary to confirm the diagno s of onychomycosis? *

*Select only one option.*

- Yes
- No

4. Do you solicit laboratory tests to confirm the diagnosis of onychomycosis? *

*Select only one option.*

- Always
- Never
- Ocasionally

5. In the case of requesting them, select which one or which (onychomycosis):

*Select as many as needed.*

- [ ] Microbiological culture
- [ ] PCR
- [ ] Histopathology

Other: [ ] ______________________

6. According to the clinical aspects observed, would you treat as onychomycosis? *

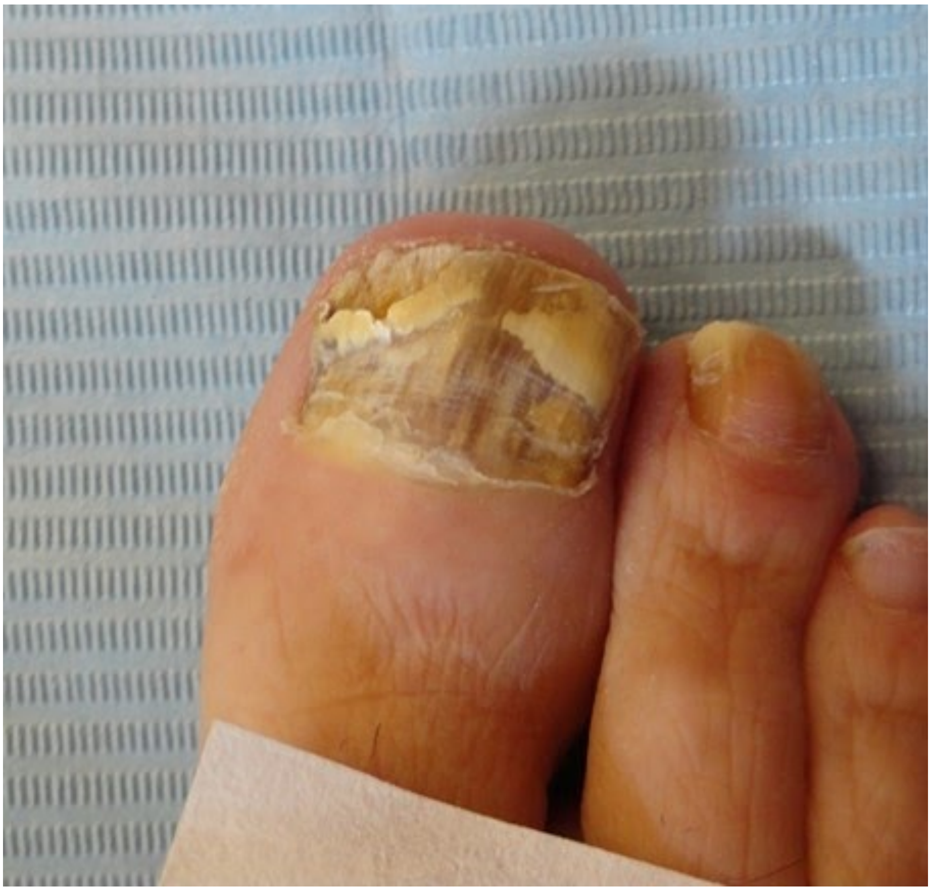

*Select only one option.*

- Yes
- No

7. According to the clinical aspects observed, would you treat as onychomycosis? *

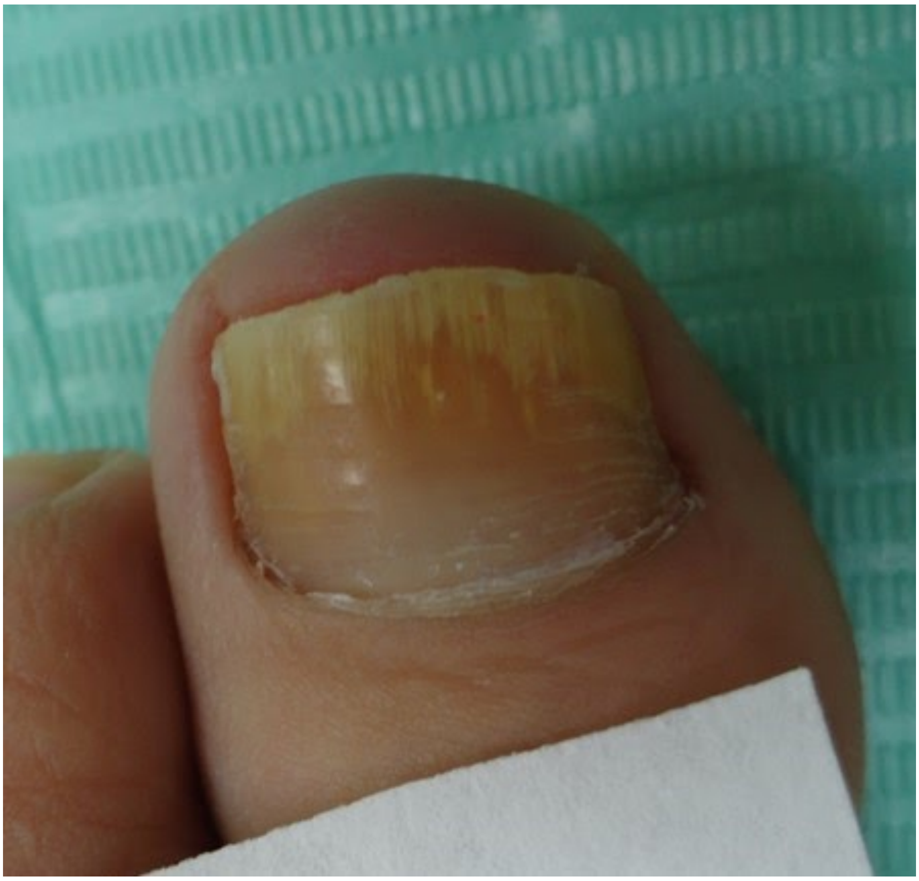

*Select only one option..*

- Yes
- No

8. According to the clinical aspects observed, would you treat as onychomycosis? *

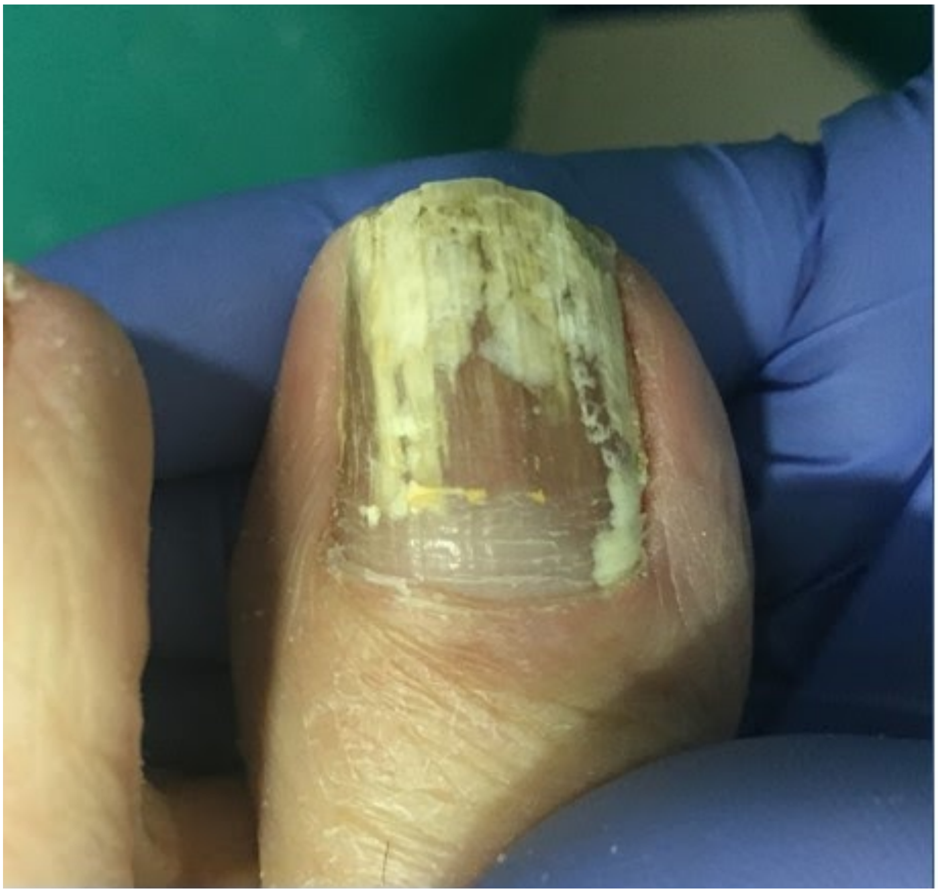

*Select only one option.*

- Yes
- No

9. According to the clinical aspects observed, would you treat as onychomycosis? *

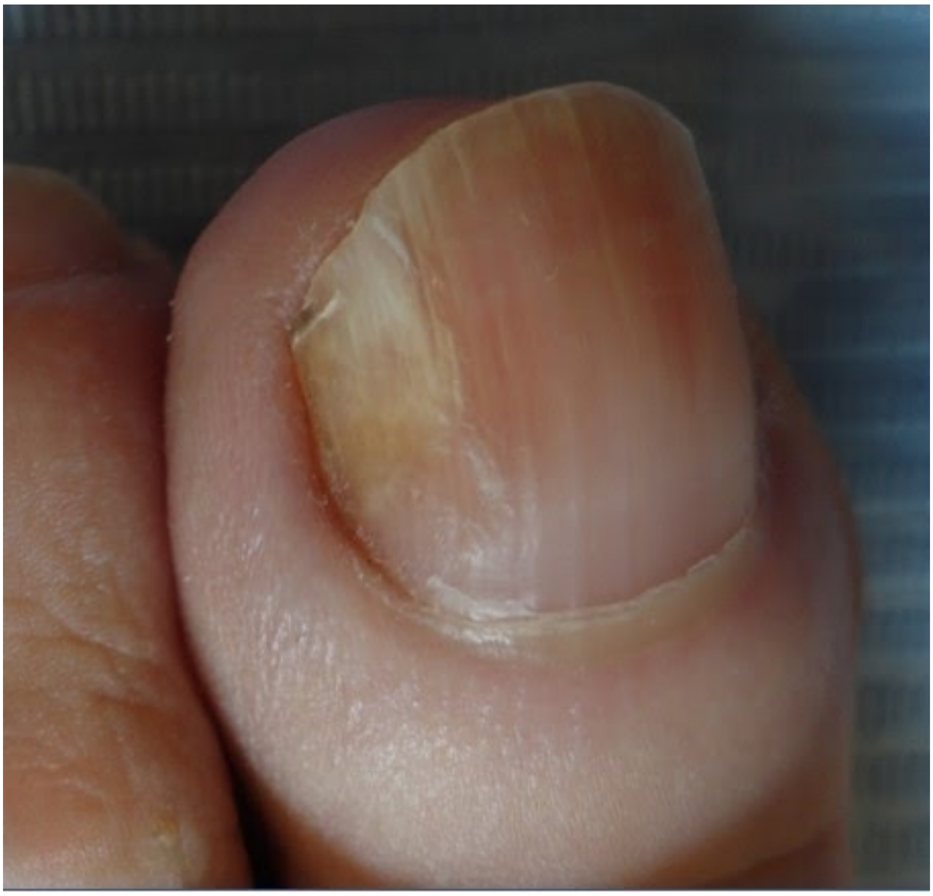

*Select only one option..*

- Yes
- No

10. According to the clinical aspects observed, would you treat as onychomycosis? *

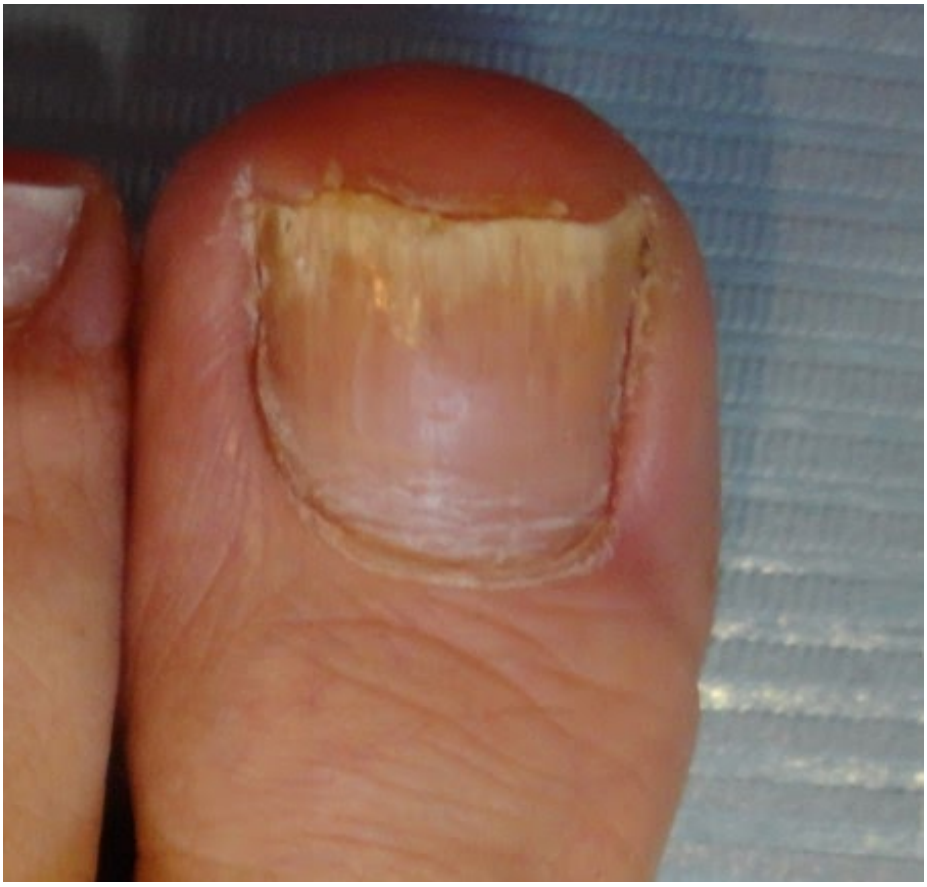

*Select only one option..*

- Yes
- No

11. According to the clinical aspects observed, would you treat as onychomycosis? *

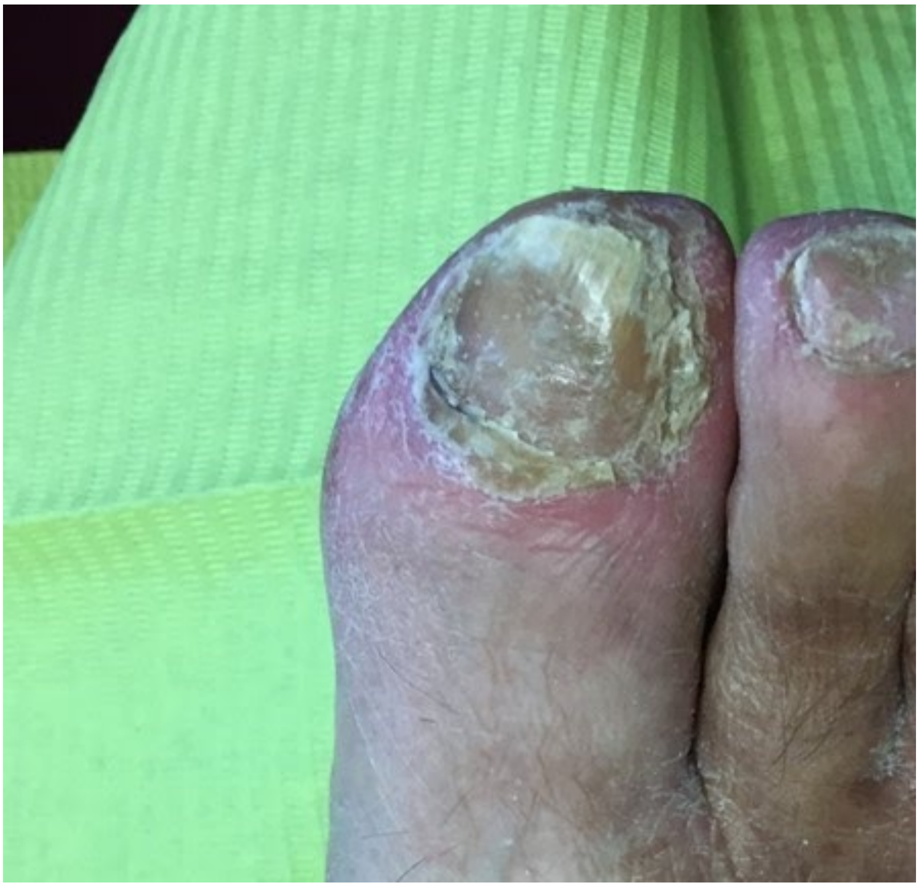

*Select only one option..*

- Yes
- No

Plantar warts.

12. Do you consider that complementary laboratory tests are necessary to confirm the diagnosis of plantar warts? *

*Select only one option.*

- Yes
- No

13. Do you solicit laboratory tests to confirm the diagnosis of plantar warts? *

*Select only one option.*

- Always
- Never
- Occasionally

14. In the case of requesting them, select which one or which (plantar warts):

*Select as many as needed*

- [ ] PCR
- [ ] Histopathology
- Other: [ ] ____________________

15. According to the clinical aspects observed, would you treat as plantar wart? *

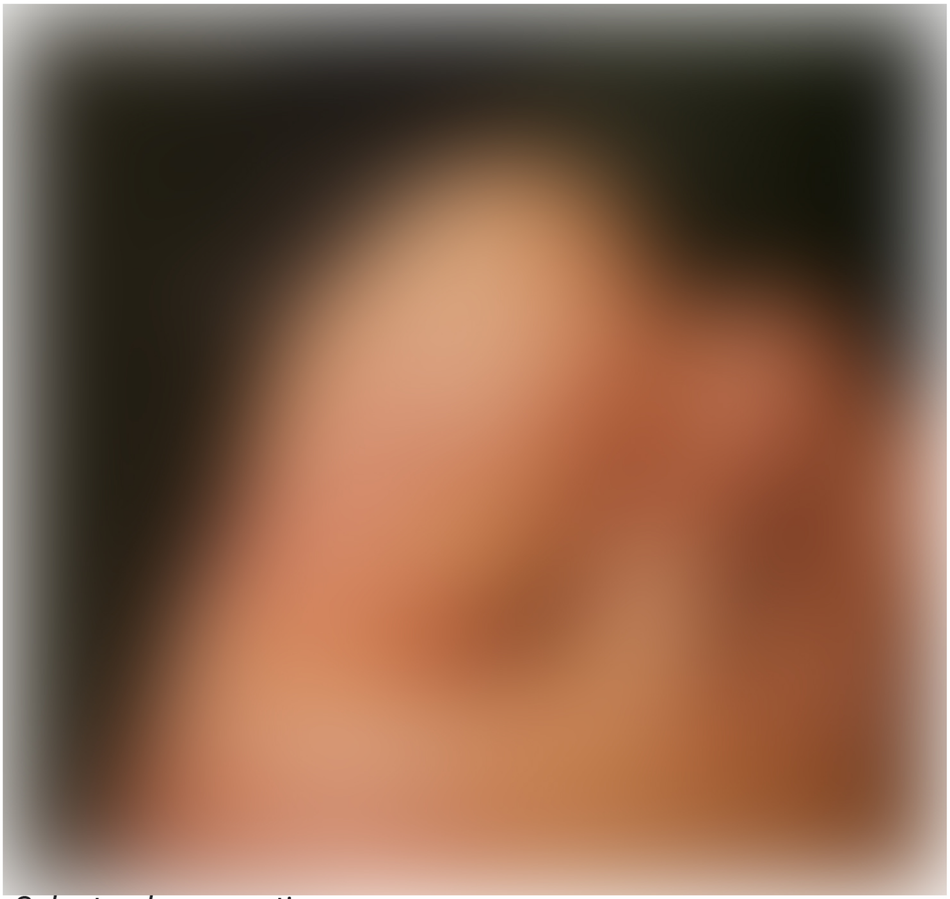

*Select only one option.*

- Yes
- No

16. According to the clinical aspects observed, would you treat as plantar wart? *

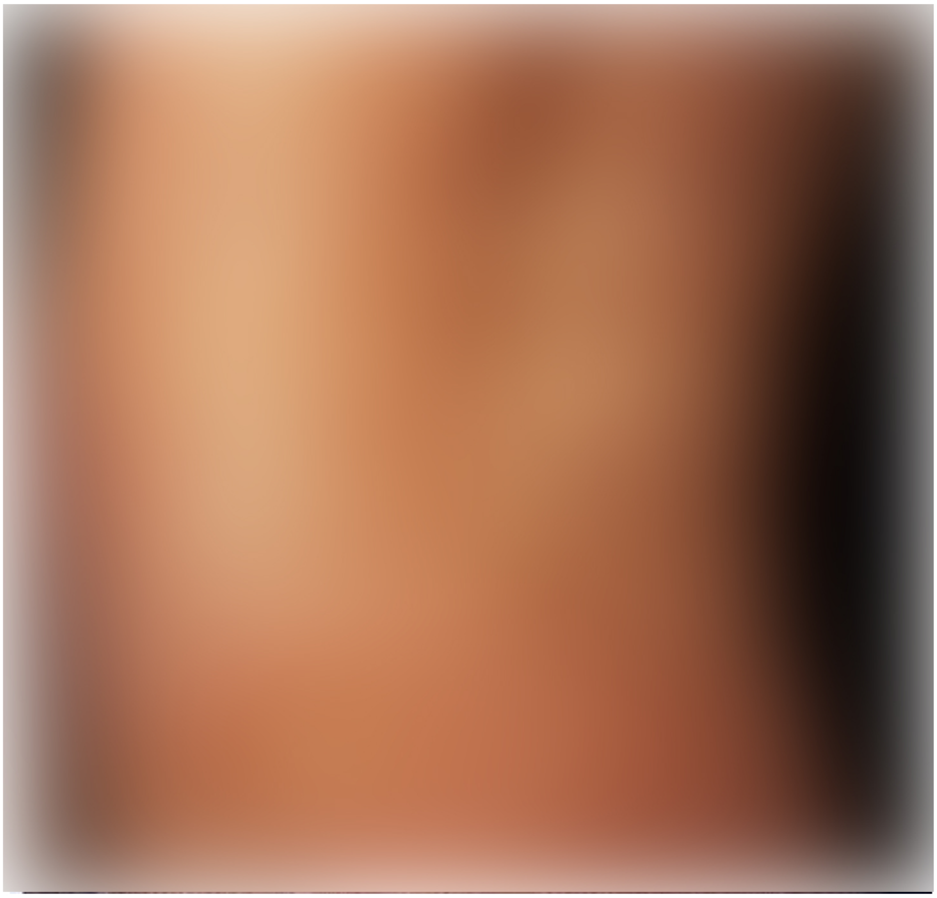

*Select only one option.*

- Yes
- No

17. According to the clinical aspects observed, would you treat as plantar wart? *

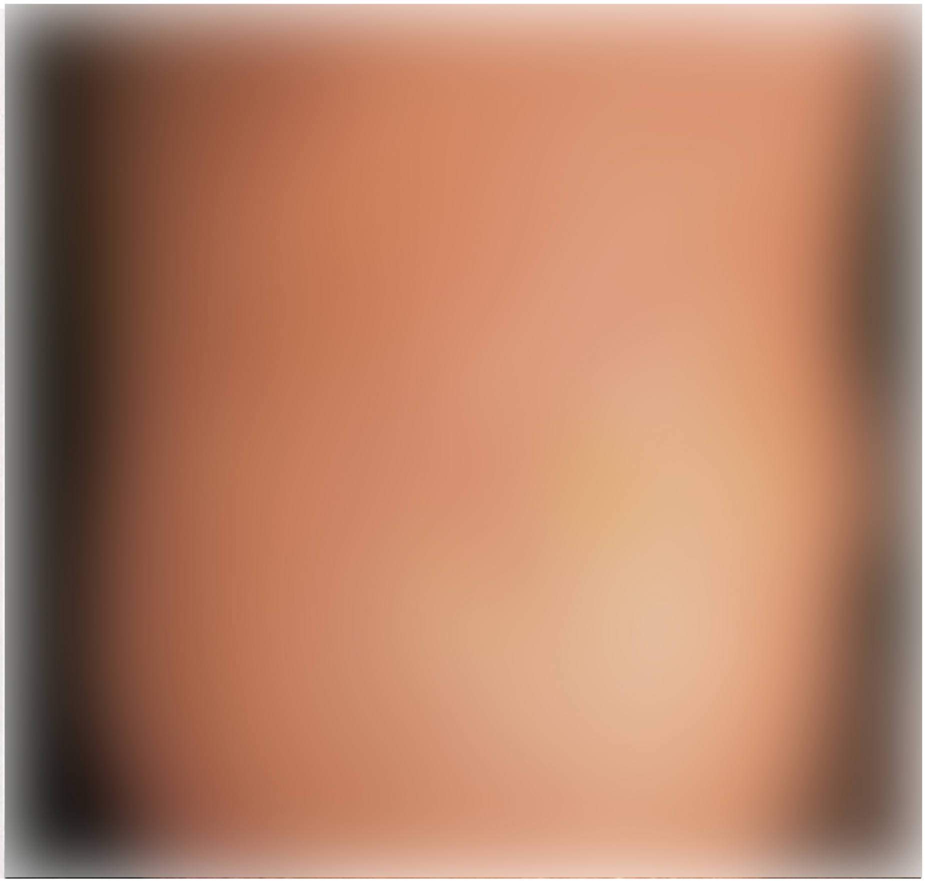

*Select only one option..*

- Yes
- No

18. According to the clinical aspects observed, would you treat as plantar wart? *

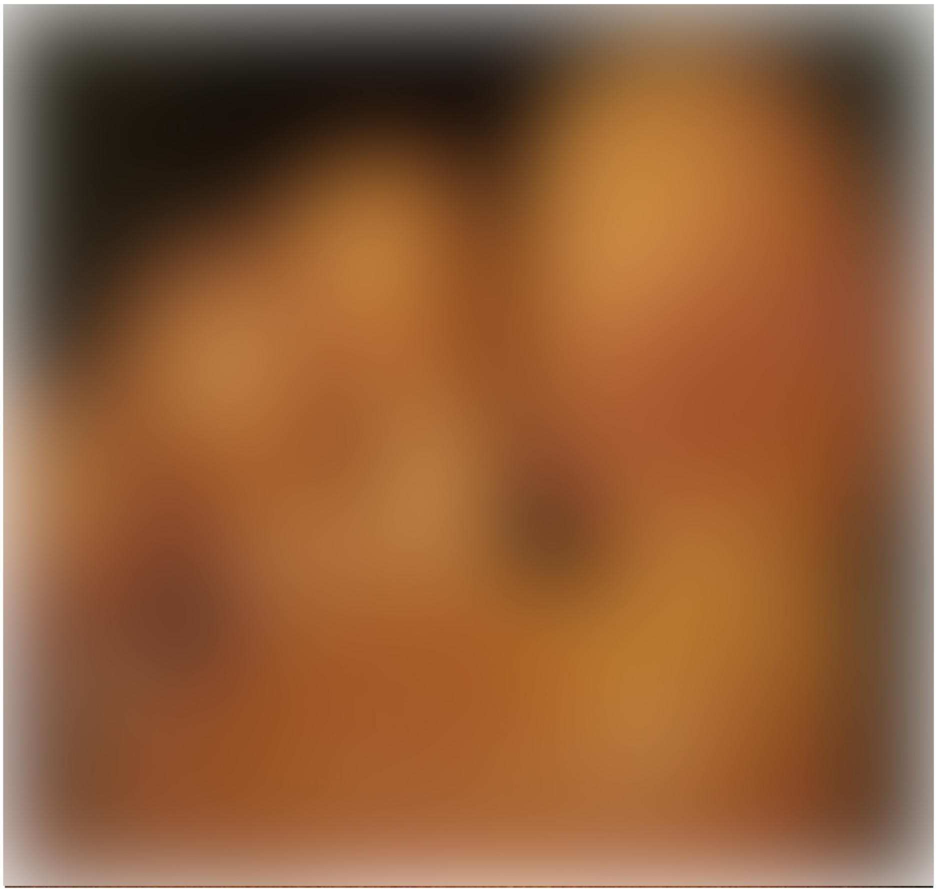

*Select only one option..*

- Yes
- No

19. According to the clinical aspects observed, would you treat as plantar wart? *

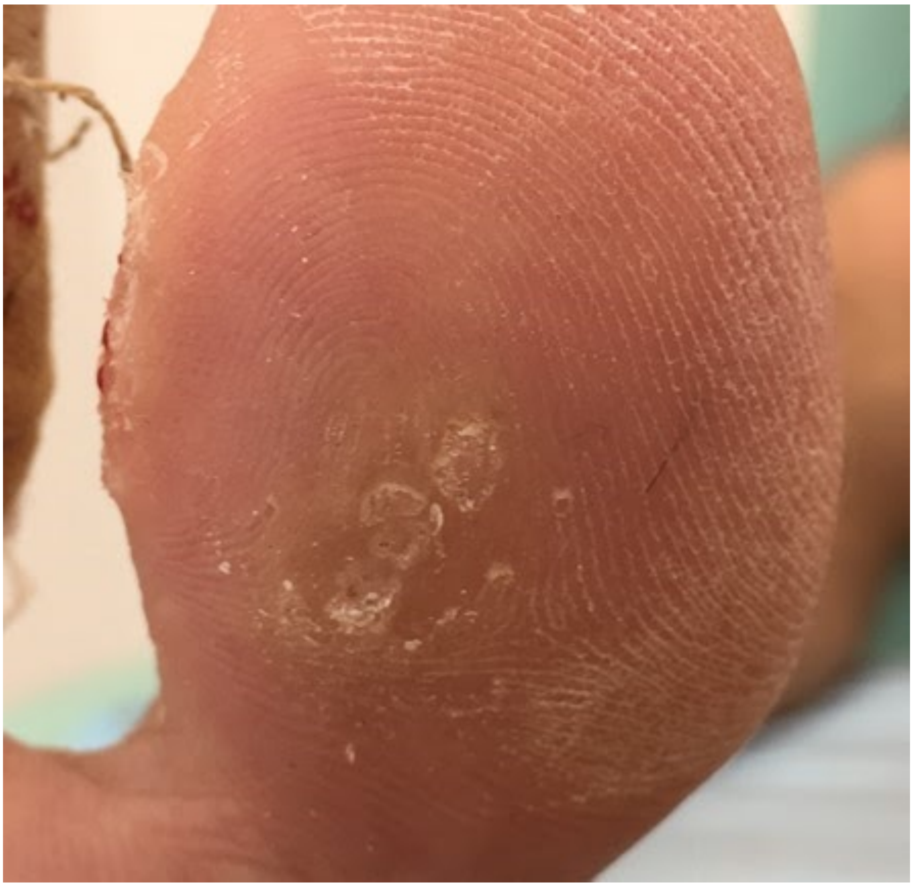

*Select only one option..*

- Yes
- No

20. According to the clinical aspects observed, would you treat as plantar wart? *

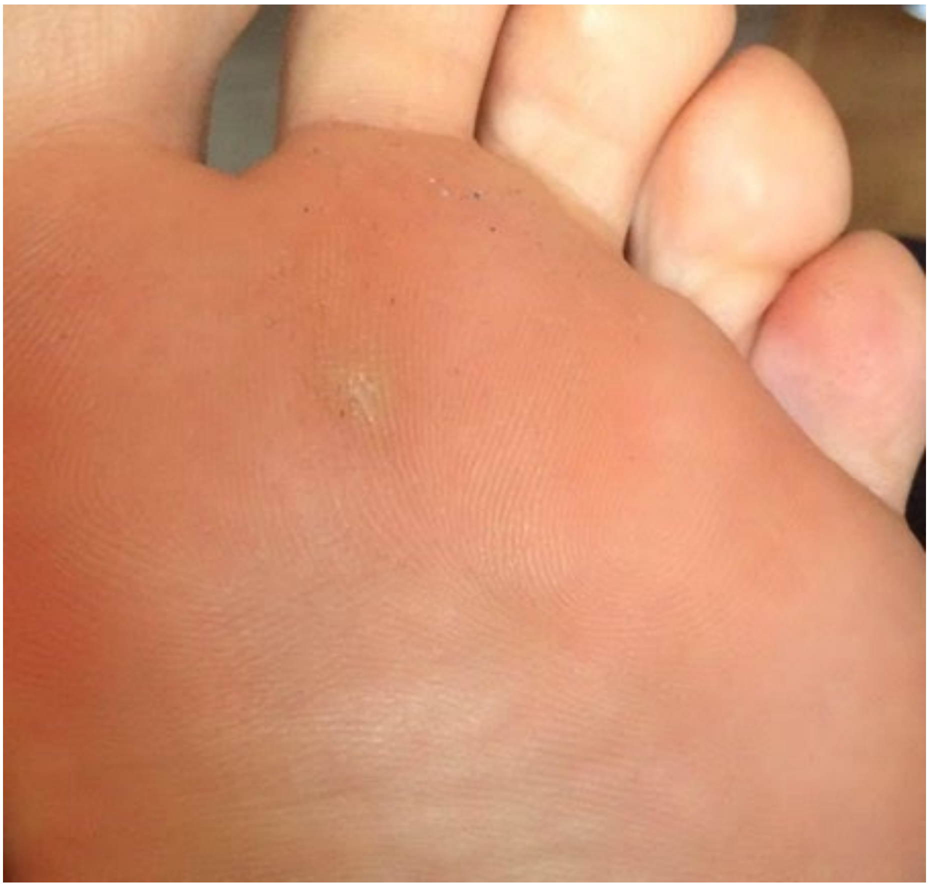

*Select only one option..*

- Yes
- No
